# T12-L3 Nerve Transfer-Induced Locomotor Recovery in Rats with Thoracolumbar Contusion: Essential Roles of Sensory Input Rerouting and Central Neuroplasticity

**DOI:** 10.3390/cells12242804

**Published:** 2023-12-08

**Authors:** Dou Yu, Xiang Zeng, Zaid S. Aljuboori, Rachel Dennison, Liquan Wu, Jamie A. Anderson, Yang D. Teng

**Affiliations:** 1 Department of Physical Medicine and Rehabilitation, Harvard Medical School, Boston, MA 02129, USA; 2 Department of Neurosurgery, Harvard Medical School, Boston, MA 02115, USA; 3 Laboratory of SCI, Stem Cell and Recovery Neurobiology Research, Department of Physical Medicine and Rehabilitation, Spaulding Rehabilitation Hospital, Mass General Brigham, Harvard Medical School, Boston, MA 02129, USA; 4 Neurotrauma Recovery Research, Spaulding Rehabilitation Hospital Network, Mass General Brigham, Harvard Medical School, Boston, MA 02129, USA

**Keywords:** nerve transfer, spinal cord injury, contusion, sensory nerve, neuroplasticity, locomotion

## Abstract

Locomotor recovery after spinal cord injury (SCI) remains an unmet challenge. Nerve transfer (NT), the connection of a functional/expendable peripheral nerve to a paralyzed nerve root, has long been clinically applied, aiming to restore motor control. However, outcomes have been inconsistent, suggesting that NT-induced neurological reinstatement may require activation of mechanisms beyond motor axon reinnervation (*our hypothesis*). We previously reported that to enhance rat locomotion following T13-L1 hemisection, T12-L3 NT must be performed within timeframes optimal for sensory nerve regrowth. Here, T12-L3 NT was performed for adult female rats with subacute (7–9 days) or chronic (8 weeks) mild (SCI_mi_: 10 g × 12.5 mm) or moderate (SCI_mo_: 10 g × 25 mm) T13-L1 thoracolumbar contusion. For chronic injuries, T11-12 implantation of adult hMSCs (1-week before NT), post-NT intramuscular delivery of FGF2, and environmentally enriched/enlarged (EEE) housing were provided. NT, not control procedures, qualitatively improved locomotion in both SCI_mi_ groups and animals with subacute SCI_mo_. However, delayed NT did not produce neurological scale upgrading conversion for SCI_mo_ rats. Ablation of the T12 ventral/motor or dorsal/sensory root determined that the T12-L3 sensory input played a key role in hindlimb reanimation. Pharmacological, electrophysiological, and trans-synaptic tracing assays revealed that NT strengthened integrity of the propriospinal network, serotonergic neuromodulation, and the neuromuscular junction. Besides key outcomes of thoracolumbar contusion modeling, the data provides the first evidence that mixed NT-induced locomotor efficacy may rely pivotally on sensory rerouting and pro-repair neuroplasticity to reactivate neurocircuits/central pattern generators. The finding describes a novel neurobiology mechanism underlying NT, which can be targeted for development of innovative neurotization therapies.

## 1. Introduction

About 18,000 people are hospitalized for spinal cord injury (SCI) each year, and currently ~299,000 individuals live with SCI disabilities in the US alone. Clinically, midcervical (C2-6) and thoracolumbar (T12-L1) injuries are the most common cases [1]. People with SCI experience a range of long-term motosensory deficits, coupled with autonomic dysfunction, systemic complications, and psychosocial impact. The need for a cure is urgent, but effective therapy for SCI is still far from reality [2,3]. Conventionally, singly focused therapeutic approaches have been investigated for SCI, including tactics to stimulate axon regeneration, supply a growth factor, and counteract a specific secondary injury event (e.g., excitotoxicity, edema, oxidative damage, neuroinflammation, reactive astrogliosis, and programmed cell death) [4,5,6]. While these research endeavors have not delivered any therapeutic candidates that have passed a phase-III clinical trial to date, they collectively demonstrated that SCI is not a monolithic entity but rather a complex of concurrent, interactive, and sequential pathophysiological courses [2,7]. Since experimental therapies mostly had certain efficacies when given preinjury or ultra-early post SCI (p.i.), but yielded a weaker effect on subacute and chronic injuries that have the highest clinical relevance, it is imperative to investigate mechanistically oriented multimodal strategies of repairing the disrupted neural/neuromuscular circuits in subacute and chronic SCI for clinical translation [3,8].

Although far less explored scientifically (e.g., modeled in mammals only), nerve transfer (NT; also termed neurotization), the surgical connection of a functional and expendable peripheral nerve (PN) to a paralyzed nerve root via end-to-end nerve anastomosis (i.e., direct coaptation), has long been tested based on the regrowth ability of the PN, and applied for treating human PN injuries for the purpose of motor axon reinnervation to reanimate the disabled muscles. NT has also been tried to partly return upper extremity or respiratory control after cervical SCI [8,9,10,11,12,13,14]. However, frequently inconsistent outcomes and complications (e.g., improper motor pattern) have hindered a routine utilization of NT protocols for managing SCI despite outcome improvement, innovative NT formulas, and new reasoning paradigms emerging in recent years [14,15,16,17,18]. We *hypothesized* that the neurotization of mixed nerves that contain both somatomotosensory axons may recruit and activate neurobiological mechanisms beyond motor reinnervation. To test it, we previously investigated an original T12-L3 NT-anchored multimodal strategy to reanimate hindlimb locomotion after subacute T13-L1 hemisection in rats. NT markedly augmented locomotion when the T12-L3 neurotization was performed in a time window permissive for sensory nerve reinnervation, which appeared crucial in generating beneficial neuroplasticity [19]. In this study, T12-L3 NT was performed for rats with subacute (7–9 days) or chronic (8 weeks) mild or moderate T13-L1 contusion (i.e., clinically relevant models of thoracolumbar SCI). For chronic SCI, the intraspinal cord implantation of adult human bone marrow-derived stromal stem cells (hMSCs) and intramuscular (i.m.) delivery of fibroblast growth factor-2 (FGF2) plus environmentally enriched/enlarged (EEE) housing were provided before or after NT intervention [19,20,21]. Besides conducting standard behavioral, histopathologic, and immunohistochemical (IHC) assays, regular and trans-synaptic neural tracing, selective ablation of the T12 ventral/motor or dorsal/sensory root, pharmacological neuromodulation, and electrophysiological evaluation were utilized to multidimensionally determine key underpinnings of the NT-reinstated hindlimb function.

## 2. Materials and Methods

### 2.1. Animals, SCI Modeling, and Perioperative Management

**Figure 1:**
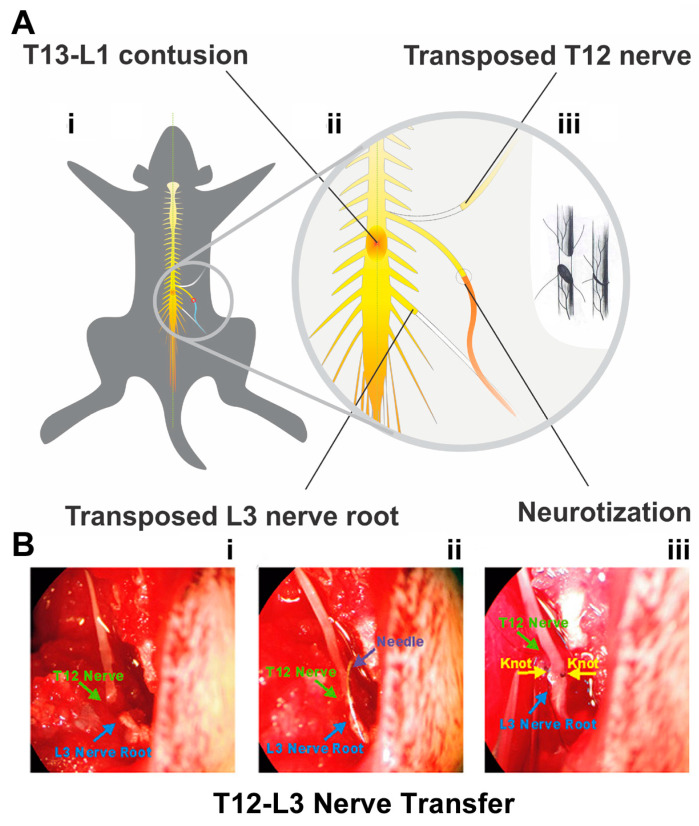
Thoracolumbar contusion and nerve transfer (NT) protocols. (**A**) The schematics demonstrate that a mild (10 g × 12.5 mm) or moderate (10 g × 25 mm) contusion to T13-L1 spinal cord was generated (**A**(**i**)) prior to performing T12-L3 nerve transfer (NT; (**A**(**ii**)) via end-to-end anastomosis (**A**(**iii**)) at 7–9 days or 8 weeks after spinal cord injury (SCI). The enlarged circle in (**A**(**ii**)) and (**A**(**iii**)) details the surgical scheme of the NT procedure regarding how the end of the T12 nerve was transposed and neurotized with the L3 nerve root. (**B**) The ends of T12 (green arrow) and L3 (blue arrow) nerves (**B**(**i**)) were anastomosed (**B**(**ii**)) by stiches (yellow arrowheads) opposite to each other (**B**(**iii**)) to complete the T12-L3 NT under a Zeiss SV6 dissecting stereomicroscope (Carl Zeiss Inc., White Plains, NY, USA).

Female Sprague Dawley rats (230–250 g; Charles River Laboratories, Wilmington, MA, USA) were housed in pairs under a 12 h light/dark photocycle in a colony under ambient temperature and humidity with food and water available ad libitum. The animal was anesthetized with i.p. ketamine (75 mg/kg) and xylazine (10 mg/kg). Following confirmation of the appropriate depth of general anesthesia, skin incision and muscle dissection were made before performing dorsal laminectomy at T12 vertebra (corresponding to the level between the T13 and L1 spinal cord) [19,22]. Gelfoam^®^ (Pfizer, New York, NY, USA) was applied for hemostasis. T13-L1 spinal cord mild (10 g × 12.5 mm) or moderate (10 g × 25 mm) contusion was generated by using the New York University (NYU) Impactor (Figure 1A(i,ii); Table 1) as per protocols described before [23,24]. Also produced was a surgical control group that received T12 laminectomy alone with bilateral transections of T12 and L3 nerves without anastomosis (i.e., sham NT) [19]. The surgical zones of the muscle and skin were then sutured (3-0 Ethilon black brand, Ethicon^®^, Sommerville, NJ, USA), and skin was stapled with wound clips (Reflex^®^; Cellpoint Scientific, Gaithersburg, MD, USA).

The proper production of SCI or laminectomy was visually confirmed by the appearance of a bruise (i.e., bilateral short dark blue lines alongside the dorsal lateral sulci) at the contusion site in all cases immediately following the weight drop, with additional validation by the standardized behavioral tests and daily checks on hindlimb paralysis, spinal reflex disorder, and bladder dysfunction [25]. The animal was individually placed in a warm cage with access to water and hydrated food pellets to recover for 12 h after surgery. The cage floor temperature was maintained by a heating pad which was irrigated by 37 °C water via an EZ-200 circulation pump (E-Z Systems Inc., Palmer, PA, USA). Lactated Ringer’s solution (ICU Medical Inc., San Clemente, CA, USA; 10 mL/day) and ketoprofen (Anafen^®^, Merial Canada Inc., Clark-Grahm Baie-D’Urfé, Québec, Canada; 5 mg/kg) were administered s.c. for 5 days for all post-surgery animals. The bladders of SCI rats were evacuated twice daily until a “reflex bladder” was established, usually around 5–9 days p.i. [24,25,26].

**Table 1:** Experimental groups.

Experimental Group	SCI Model or Laminectomy	N	T12 Intraparenchymal Implantation of hMSCs	T12-L3 Nerve Transfer (NT)	FGF2 Injection (i.m.)	EEE Cage Housing
1. LAM/NT_sham_	T13-L1 Laminectomy and sham NT (control)	12	None	Bilateral, n = 12	None	No
2. SCI_mi_	Mild contusion (10 g × 12.5 mm) only (control)	14	None	None	None	Starting 1 week post SCI for 4 weeks
3. NT_sami_	Mild contusion with subacute (7–9 days p.i.) T12-L3 NT	14	None	Unilateral, n = 7 Bilateral, n = 7	None	No
4. NT_dmi_	Mild contusion with delayed (8 weeks p.i.) T12-L3 NT	8	Injection of 4 µL hMSCs (5 × 10^4^ cells/µL) per rat done 7 weeks p.i.	Unilateral, n = 3 Bilateral, n = 5	Administration of 0.8 μg FGF2 per rat in the quadriceps	Starting 1 week post NT for 4 weeks
5. SCI_mo_	Moderate contusion (10 g × 25 mm) only (control)	18	None	None	None	Starting 1 week post SCI for 4 weeks
6. NT_samo_	Moderate contusion with subacute (7–9 days p.i.) T12-L3 NT	14	None	Unilateral, n = 7 Bilateral, n = 7	None	No
7. NT_dmo_	Moderate contusion with delayed (8 weeks p.i.) T12-L3 NT	12	Injection of 4 µL hMSCs (5 × 10^4^ cells/µL) per rat done 7 weeks p.i.	Unilateral, n = 7 Bilateral, n = 7	Administration of 0.8 μg FGF2 per rat in the quadriceps	Starting 1 week post NT for 4 weeks
8. Naïve	None	7	None	None	None	No

Notes: (1) NT: nerve transfer. SCI: spinal cord injury. LAM: laminectomy. i.m.: intramuscular. SCI_mi_: mild contusion. hMSCs: human mesenchymal stromal stem cells. SCI_mo_: moderate SCI. NT_sami_: subacute NT for SCI_mi_. NT_dmi_: delayed NT for SCI_mi_. NT_samo_: subacute NT for SCI_mo_. NT_dmo_: delayed NT for SCI_mo_. p.i.: post injury. (2) Comparison between NT_sami_ and NT_samo_ vs. SCI_mi_ and SCI_mo_, respectively, was justified as earlier work showed no significant impact of a similar EEE housing formula on subacute NT for SCI rats [19]. (3) Since hMSC debris or vehicle as a control treatment did not produce any discernible effect [20,27], they were not used to set up specific control groups.

### 2.2. Experimental Design

Animals, surgeries, behaviors, and statistical power analysis. Habituation for ≥3 days was provided for all animals, during which the baseline physical conditions and behaviors were examined. For SCI modeling, female rats have been conventionally chosen for their less frequent contraction of lower urinary tract (LUT) complications under standard post-care [23,27]. The surgeries, behavioral tests, and other investigative procedures were conducted largely following a randomized block design. Behavioral tests were performed by two researchers who were blinded to the treatment. At the end of the study, euthanasia and tissue fixation were performed to collect the spinal cord, brain, PN, and muscles [20]. The experimental procedures were carried out in strict accordance with the Guide for the Care and Use of Laboratory Animals (US National Research Council Committee for the Update of the Guide for the Care and Use of Laboratory Animals, 8th edition) after review and approval by the Institutional Animal Care and Use Committee (IACUC) of Harvard Medical Area (HMA) Standing Committee on Animals or VA Boston Healthcare System IACUC.

The power analysis results of our earlier studies were used to estimate the sample size. Based on the outcome measures obtained from similar SCI models, it was determined that with 7 rats per group, there is an 87% probability of detecting an effect ≥50% in ventral horn (VH) neuronal sparing, whereas an 80% probability exists for detecting an effect ≥47% in intermediolateral (IML) neuronal sparing at a spinal level 3 mm caudal to the injury epicenter [27,28]. With 12 rats per group, there is an 80% probability of detecting an effect ≥34% in inclined plane degree, 25% in the hindlimb combined behavioral score, and 15% in the white matter (WM) area at the epicenter [29,30]. Finally, with the α value of 5%, an average sample value of 0.41 g (post-SCI sensory response threshold), mean test value (post-treatment) of 3.1 g, and group size of 4 rats, the statistical power (i.e., 1-β) equals ~100%, indicating that the β value ≈ 0 [31].

Experimental groups. According to a blocked design, animals were randomly assigned to 7 experimental groups that are exhibited in Table 1 after the laminectomy and/or contusion consistency was verified. Laminectomy plus sham NT (Group 1), SCI alone (Groups 2 and 5), and naïve (Group 8) animals were included as controls. Groups 4 and 7 were given i.m. FGF2 immediately after T12-L3 NT [19,28,32]. Animals in Groups 2, 4, 5 and 7 were housed in EEE cages for 4 weeks, starting 1 week post NT [19]. With team assistance, all surgeries were performed independently by two investigators in ~30–70 split (D.Y. and X.Z.); the statistically indiscernible outcomes were combined for the final data presentation.

### 2.3. Thoracic 12 (T12) Intercostal Nerve and Lumbar 3 (L3) Nerve Root Transfer

At either 7–9 days or 8 weeks p.i., T12-L3 NT was performed via end-to-end nerve anastomosis (Figure 1A(iii)) in rats under general anesthesia. Following our standard protocol [19], the T12 nerve coursing between the T12 and T13 thoracic ribs was partly dissected and cut at the point of the bifurcation. The distal end of the T12 nerve was freed and the adjacent nerve trunk maintained moist between muscles covered with a saline-soaked gauze. Next, the L3 transverse process was dissected and removed to expose the L3 nerve root, which was transected before it branched to ensure the neurotization of the T12 nerve end with the entire distal L3 nerve. The ends of T12 and L3 nerves were anastomosed with stitches opposite to each other (10/0 nylon sutures, Ethicon, Sommerville, NJ, USA) under a Zeiss SV6 dissecting stereomicroscope (Carl Zeiss Inc., White Plains, NY, USA) (Figure 1B). Special efforts were given to sewing through the outer layer of the nerve tissue only to avoid pinching of the neurotized nerve ends. In the LAM/NT_sham_ group (Table 1), the dissected T12 and L3 nerve ends were not anastomosed.

### 2.4. Human MSC (hMSC) Implantation

For the NT_dmi_ and NT_dmo_ groups (Table 1), administration of Prograf^TM^ (Astellas Pharma US Inc., Northbrook, IL, USA; 1 mg/kg/day, in drinking water) began 2 days before hMSC (from NIH) [20] implantation and continued for the rest of study. At 7 weeks p.i., using the same general anesthesia protocol, skin preparation, tissue dissection, and pre-/post-care, incision of the skin over the T10-11 vertebrae and laminectomy of the caudal T10 and rostral T11 dorsal plates were made (length: 3 mm). A total volume of 4 µL suspension of hMSCs (5 × 10^4^ cells/µL × 4 = 2 × 10^5^ cells/rat) was stereotaxically injected into 3 loci adjacent to the dorsal central sulcus (1 µL/rostral, 2 µL/middle, and 1 µL/caudal; 1 mm apart; zone length: 2 mm; depth: 1 mm) of the T11-12 parenchyma [20,33]. Each injection lasted 2 min, and the tip of the glass pipet remained inside the parenchyma for another 2 min before a slow removal to prevent backflow of the cells [34].

### 2.5. Administration of FGF2

Per findings by Konya et al. (2008) [19], animals in groups NT_dmi_ and NT_dmo_ received FGF2 (also called basic FGF: bFGF) unilaterally (for unilateral NT) or bilaterally (for bilateral NT) in the quadriceps femoris muscle (also termed the quadriceps: innervated by L2-L5 PNs) [35,36] immediately after T12-L3 NT, taking advantage of the intra-nerve retrograde transportation capacity of FGF2 to augment PN regrowth [37]. Under the same general anesthesia, the quadriceps were surgically exposed. Four pieces of Gelfoam^®^ (Pfizer; 1 mm^3^) soaked with FGF2 solution (Calbiochem, San Diego, CA, USA; 10 μg/mL; ~20 μL or ~0.2 μg/per piece) were embedded within the muscles at four different loci near the femoral nerve roots (total dose: ~0.8 μg FGF2/rat) [19].

### 2.6. EEE Housing

Animals in the NT_dmi_, SCI_mi_, NT_dmo_, and SCI_mo_ groups were kept in large double-floor plexiglass cages (30″ × 20″ × 18″ = 10,800 in^3^/EEE cage; 4–6 rats/per cage, compared to a conventional shoebox cage: 16″ × 8″ × 8″ = 1024 in^3^/cage; 2 rats/per cage; Figure S1) for 4 weeks, starting 1 week after receiving delayed NT or SCI (Table 1). The EEE cage was designed by the PI’s lab and custom manufactured to stimulate physical activity via placing short tunnels, increasing distance of ambulation by providing additional space and keeping ad libitum food and water supplies separately at opposite ends and different floors, and enhancing locomotor intensity through a stair climbing setup (Figure S1). These arrangements can jointly augment environmental stimulation, social interaction, and physical exercise [19,38].

### 2.7. Pharmacological Verification of Neuromodulation

To evaluate the role of descending serotonergic neuromodulation of the raphespinal tract (RpST) in the post-NT hindlimb locomotor change, varied single or combinatorial pharmacological manipulations of serotonergic neurotransmission were conducted for representative rats in the NT_sami_ group at ≥12 weeks p.i. (Table 2). This group had marked post-NT neurobiological improvement that could be more readily affected by the designed drug assays. The treatments were sequentially administered to each rat (n = 5/each drug) with ≥48 h intervals (i.e., ≥5 × T1/2 of the drugs) [25,39,40,41].

### 2.8. Dorsal Rhizotomy and Ventral Root Transection

To assess the role of the sensory input from the NT-constructed T12-L3 nerve in hindlimb locomotor improvement in NT_sami_ animals, T12 dorsal root rhizotomy was performed 14–16 weeks after T12-L3 NT. NT_sami_ animals had the highest level of hindlimb recovery after NT, which would be more responsive to a new PN lesion if the ablated dorsal root were a major mediator of the NT-improved locomotion. Conversely, NT_samo_ animals showed more limited functional improvement, and ventral root ablation would be more effective to cancel the recovered portion of the locomotor ability should this nerve have played a pivotal role in channeling this neural repair (note: these procedures should be tested in animals that have experienced the same model of SCI and NT treatment in the future). T12 ventral root (mainly containing motor axons and some sensory fibers) [42] transection was performed for NT_samo_ animals (n = 4/each group). Briefly, after standard pre-operation and anesthesia preparations, the animals underwent T11 laminectomy and T12-13 partial lateral laminectomy and limited durotomy to expose the T12 dorsal or ventral root. Vannas Spring Scissors (cutting edge: 2 mm; tip diameter: 0.05 mm; Fine Science Tools, Heidelberg, Germany) were used to sever the T12 dorsal root at a locus proximal to the dorsal root ganglion (DRG), or ventral root under a Zeiss SV6 surgical stereomicroscope. In either case, the transection created a small gap to prevent spontaneous PN regeneration. The rhizotomy was independently verified by another researcher. After completing hemostasis, dura suture (Ethicon 10-0), and wound closure, animals were returned to recover according to the post-care protocol afore-described.

### 2.9. Behavioral Evaluations

A battery of comprehensive behavioral tests was started at 1–3 days before SCI, followed with assessments performed 1 day after surgery and weekly thereafter to measure hindlimb functions.

Coordinated hindlimb functions. (1) Open-field locomotion test: The general ambulatory ability was examined by placing each animal on a 50″ × 30″ dark rubber mat with a patterned surface texture to explore without disturbance. The Basso, Beattie and Bresnahan (BBB) Scale, which ranges from 0 to 21 (i.e., 0: no observable hindlimb movement; 21: a normal performance), was used to quantify the level of hindlimb locomotor function as reported before [43]. Based on the previous observation that the reconnection failure of subacute T12-L3 NT prevented the BBB score enhancement during weeks 2 to 4 post NT in rats with T13-L1 hemisection [19], SCI animals that failed to exhibit BBB score improvement during the same timeframe were not included for further data analysis (~1–3 rats in each group; note: the failure of T12-L3 anastomosis was also verified by performing a brief post-euthanasia examination).

(2) The incline plane test: This test measures the animal’s ability to maintain stable body position when positioned head-up and head-down (i.e., the rostrocaudal axis of the trunk is parallel to the longitudinal axis of the plane). The functional status was quantified by the highest angle of incline at which an animal could maintain its posture for 5 s [23,26].

(3) Footprint analysis: Utilizing a modified version of the apparatus we previously used—i.e., a 40″ × 3.2″ track with both sides boarded up (height: 14″), which was lined with a continuous white paper roll [19]—representative rats (i.e., with BBB scores most close to the group mean) were first acclimated and trained to ambulate straightforwardly through the path. To collect the footprint, paws of the trained animals from each group (i.e., Naïve control, SCI_mi_, NT_sami_, and NT_dmi_) were differentially ink-colored (i.e., red: hindpaw without NT; blue: hindpaw with NT; yellow: both forepaws) and verified for print quality. The animals were then individually released to walk through the paper-lined track with the footprints being later scanned to produce the digitized images for further analysis. A series of established footprint parameters were generated (i.e., PL: paw print length; TS: toe spread; and IT: intermediate toe spread) to assess the levels of hindlimb dysfunctional severities [19,44].

Sensory functions. To minimize the experiences of animals for more lengthy test procedures such as those of von Frey filament test in order to prevent disruption of the neurotized T12-L3 nerve, only two well-established spinal sensory reflex tests were used [29,30]. Hindlimb withdrawal reflexes to brief stimulations of pinch/nociception and pressure were tested to measure sensory changes in different experimental groups. The response levels were scored according to our published system (i.e., 0: areflexia; 1: hyporeflexia; 2: normoreflexia; or 3: hyperreflexia) [26,31]. To further differentiate the amplitude of the response, scores of 0.5, 1.5, and 2.5 were added to depict outcomes with speed and scale between 0–1, 1–2, and 2–3, respectively. Among all the scores, 2 was considered normal and all other scores abnormal. In addition, these tests were not performed for the SCI_mo_ animals to mitigate physical interferences.

### 2.10. Electrophysiological Evaluations

Motor-evoked spinal cord potentials. Anesthetized rats from NT_sami_ and SCI_mi_ groups were prepared for electrode placement in the primary somatomotor cortex (M1 or SMC) —the layer-5 pyramidal motor neurons (MNs) —via a Kopf stereotaxic frame (coordinates: Bregma: −1.5 mm; mediolateral: 1.4 mm; and depth: 1.0–1.5 mm) [45,46,47]. The cortical side was contralateral to the T6 and L3 dorsolateral CST recording loci (0.1 mm lateral to the dorsal central sulcus and 0.8 mm below the dorsal surface). Bipolar electrical stimulations (10–3000 Hz/signals filtering bandpass frequency; 0.1 ms/pulse width; 0.05–10 mA/intensity; 15 Hz/repetition rate) were provided for a recording electrode placed alternately at L3 (below-injury level) and T6 (above-injury level) to collect the cerebral motor-evoked potentials (CMEPs), with each recording session of 10–15 min [48,49].

Segmental spinal cord pathway integrity. For assessing the functional integrity of the spinal cord pathway in NT_sami_ and SCI_mi_ animals, electrical stimulations were given via an electrode placed on the C7 spinal dorsal root (10–3000 Hz/bandpass; 100 µs/pulse width; 10 mA/intensity; 70 Hz/repetition rate) and a recording electrode in the T11 intermediate gray matter (IMG) in Rexed Lamina VII (RL-VII: 0.7 mm lateral to the dorsal central sulcus and 0.8 mm below the dorsal surface) [49] to measure spinal cord stimulation-evoked potentials. This range of the spinal cord contains both the cervical/long and thoracic projection propriospinal networks [50,51].

### 2.11. Regular and Trans-Synaptic Neural Tracing

After functional evaluations, animals were anesthetized and stabilized on a Kopf stereotaxic frame. Selected tracers were injected into the SMC, spinal cord parenchyma, and target muscles to label the CST and T12-L3 NT-related nerves, neurons, neuronal network (e.g., the propriospinal projection circuit), and neuromuscular junction (Table 3) [19,20,45]. The following tracers were used: (1) 10% (wt/vol solution) biotinylated dextran amine (BDA; Molecular Probes Inc., Eugene, OR, USA) in phosphate-buffered saline (PBS): an anterograde tracer [45]; (2) 2% (wt/vol solution in PBS) FluoroGold (FG; Fluorochrome LLC., Denver, CO, USA): a retrograde tracer [19]; (3) DiI crystals (Molecular Probes Inc.): a fluorescent lipophilic dye that diffuses along the lipid bilayer of membranes, traveling retrogradely or anterogradely [20]; and (4) 2% (wt/vol solution in PBS) WGA-Alexa 488 (Life Technologies, Carlsbad, CA, USA): a trans-synaptic tracer [52]. Animals were maintained for 4–8 weeks before euthanasia and tissue collection. BDA signal was revealed on mounted or floating 20 or 30 μm spinal cord and 40 μm brain coronal sections with a Vector Elite ABC kit (Vector Laboratories Inc., Newark, CA, USA) and DAB kit (Pierce Biotechnology Inc., Rockford, IL, USA), or 20 µg/mL Fluorescein Avidin DCS (Vector Laboratories Inc.). FG, DiI, or WGA-Alexa 488 signals were observed directly or with IHC detection of neurofilament-H (NF-H) [19].

### 2.12. Neuromuscular Junction Labeling with α-Bungarotoxin

Tissue sections of the quadriceps and the quadratus lumborum collected from rats randomly selected from the NT-treated and SCI control groups (3 sections/rat, n = 3/group) were stained with α-bungarotoxin (Molecular Probes Inc.) to visualize the NMJ. The muscles were sampled from post-euthanatized animals (see below) and fixed in 4% PFA for 30 min before cryostat sectioning (30 µm). After PBS rinsing (20 min) the tissue was immersed in PBS containing 0.1 M glycine. Sections were then room temperature-incubated for 30–50 min with rhodamine-conjugated α-bungarotoxin (1:200 in PBS; Molecular Probes Inc.; Table 4) to label the α subunit of the nicotinic acetylcholine receptors (AChRs) of NMJs [19,53]. The NMJ profiles were compared to those in the same muscle groups of naïve rats (n = 2).

### 2.13. Tissue Preparation

Animals were euthanatized with i.p. 90 mg/kg ketamine and 15–20 mg/kg xylazine followed with intracardiac perfusion of 0.1 M phosphate buffer (PB; pH 7.4) and 4% PFA (paraformaldehyde in 0.1 M PB; pH 7.4). Afterward, spinal cords, brains, peripheral nerves, and muscles were collected for overnight post-fixation in 4% PFA and sequential dehydrations in 10–30% sucrose solutions prior to freezing at −50 °C in 2-methylbutane/isopentane (Sigma-Aldrich, St. Louis, MO, USA) for −80 °C storage. The neural tissues were embedded in Tissue-Tek OCT compound (Sakura Finetek, Torrance, CA, USA) and cryostatted to generate either circulation series of 20 μm transverse slices or 30 µm longitudinal sections. Longitudinal spinal cord sections were cut starting from the ventral side. Brain tissues were sectioned into 40 μm coronal slices series. The slide mounting of the tissue sections was arranged according to our established formulas [19,26,31].

### 2.14. Histopathology

Briefly, selected tissue section slides of each animal were stained with solvent blue (Sigma-Aldrich) plus hematoxylin and eosin (H&E; Sigma-Aldrich) as per our standard protocols [26,31]. Representative anastomosed nerve junctures were carefully dissected, removed, and placed on a microslide for whole-mount microscopy after IHC reaction of NF-H. The histochemical stains were imaged through an Axiovert 200 microscope with a digital Axiocam camera (Carl-Zeiss Microimaging). Morphological analysis of tissue was computed through ImageJ^®^ (ImageJ2; National Institutes of Health) to characterize histopathological outcomes [20,31].

### 2.15. IHC Assays

Standardized IHC assays were conducted on proper tissue sections to detect specific molecular markers [19,26,31]. Concisely, the control tissue-paired sections were quickly washed, hydrated, and perforated using PBS with 0.03% Triton X-100 (PBST; Sigma-Aldrich) before applying the blocking solution (4% vol/vol normal donkey serum; Jackson ImmunoResearch Laboratories, West Grove, PA, USA) in PBST for 1 h at room temperature. Selected primary antibody solutions (Table 4) were then given for an overnight reaction at 4 °C followed by PBST washing and a 1 h incubation under room temperature with the appropriate secondary antibody (Table 4). After washing, the slides were cover-slipped with Vectorshield^®^ Anti-Fade Mounting Medium with DAPI nuclear counterstain (Vector Laboratories Inc.) for evaluation.

Specific IHC markers were analyzed through confocal imaging via a Nikon C2 Laser Scanning Confocal Microscope equipped with NIS-Elements Software 4.30.1 (Nikon Inc., Melville, NY, USA). The orthogonal optical slices were reconstructed to z-stack 3D images of 10 μm thickness consisting of 1 μm steps by using NIS-Elements (Nikon Inc.) to confirm immunoreaction specificity. Each marker’s positive fluorescent threshold range was computed by averaging the pixel brightness of the weakest positive labeling signals and that of the strongest signals against the average background luminance level [26]. Quantification of positive IHC pixels for each marker was performed with ImageJ2^®^ to generate the % of the area that contained qualified signals of each antigen, i.e., immunoreactivity level, against the visual field comparably selected for all samples.

### 2.16. Statistical Analysis

SPSS^®^ software version 19 (IBM Corp., Armonk, NY, USA) or GraphPad Prism (version 7.0, GraphPad Software, San Diego, CA, USA) were used for all statistical analysis computations. Data of behavioral tests, histopathological quantifications, and IHC pixel semi-quantification were analyzed by two-way repeated-measures ANOVA or one-way ANOVA, followed with Tukey’s post hoc test when the difference between two study groups of either unequal or equal sample sizes was determined. Values were expressed as mean ± S.E.M. (the standard error of the mean). For all outcomes, statistical significance was set at *p* < 0.05 [19,26,31].

## 3. Results

The two types of contusion injuries caused transient and inconsequential bodyweight loss (i.e., 1–7% of the pre-surgery level), which recovered within 3–7 days after the surgical procedure. Other surgeries only triggered negligible bodyweight reductions. No significant differences in bodyweight changes were observed between experimental groups (*p* > 0.05, ANOVA). Further, there were no discernible adverse effects (e.g., abnormal behaviors, hyperalgesia, self-injurious behavior, etc.) resulting from the treatment of NT, FGF2, and/or neural tracers.

### 3.1. Hindlimb Motor Dysfunction following T13-L1 Contusion

**Figure 2:**
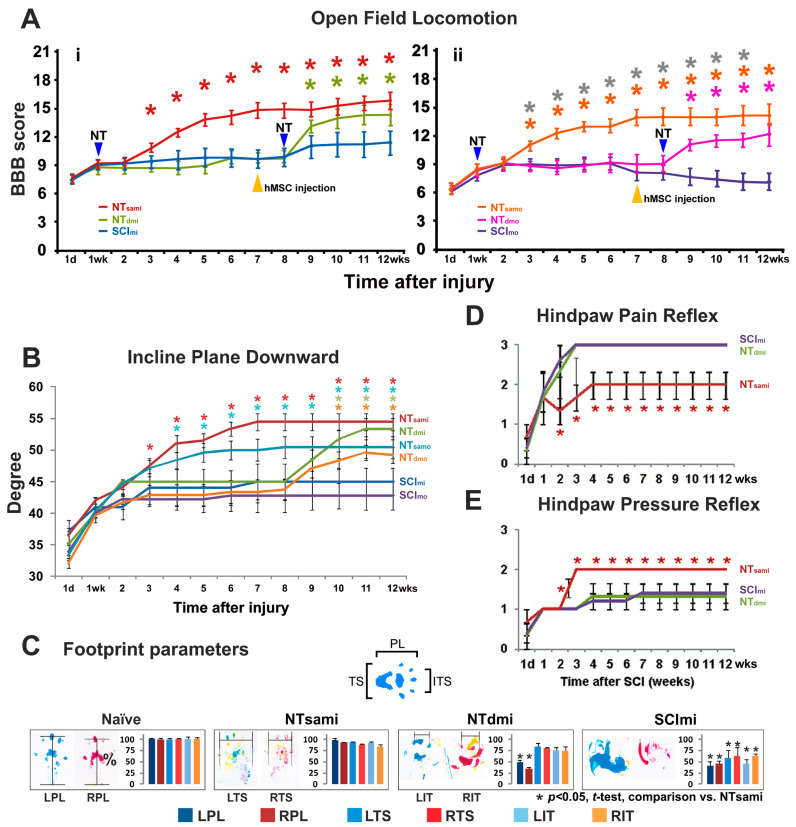
Behavioral outcomes after T13-L1 contusion and T12-L3 NT. (**A**) Locomotion. All spinal cord injury (SCI) animals with mild or moderate T13-L1 contusion displayed more serious loss of hindlimb locomotion at 1 day post injury (p.i.; group average BBB score: 7.5 and 6, respectively). In SCImi rats (n = 14), limited functional amelioration emerged afterward (**A**(**i**)). SCI_mo_ rats (n = 18) showed an even weaker natural recovery (**A**(**ii**)). By 2 weeks post NT, NT_sami_ rats exhibited locomotion enhancement, and started having bodyweight-bearing hindlimb stepping with ipsilateral forelimb coordination one week later (mean BBB score: ≥12; n = 14; (**A**(**i**)). NT_samo_ animals (n = 14) showed a similar locomotor recovery pattern (**A**(**ii**)). Both groups performed significantly better than the SCI_mi_ and SCI_mo_ control groups (*, *p* < 0.05, two-way repeated measures ANOVA with Tukey’s post hoc test; color stars: NT vs. SCI; grey stars: NT_samo_ vs. NT_dmo_). While delayed NT and i.m. FGF2 release, given 8 weeks after the SCI and 1 week following near epicenter hMSC injection (plus EEE housing), also significantly benefited locomotor function of NT_dmi_ (n = 8) and NT_dmo_ (n = 12) groups (**A**(**i**,**ii**)); *p* < 0.05), it did not induce BBB scale upgrading conversion in NT_dmo_ animals (i.e., average BBB scores: <12; (**A**(**ii**)). (**B**) Incline plane. The SCI control animals showed a trend of impairment in their stability on an inclined plane, which was similar to that of locomotion. When positioned facing downward, performance was significantly improved in NT_sami_ and NT_samo_ groups compared to corresponding controls (*, *p* < 0.05; two-way repeated measures ANOVA with Tukey’s post hoc test). On a lesser scale, the group mean angle of NT_dmi_ and NT_dmo_ rats was also significantly improved starting 2 weeks and 3 weeks after neurotization, respectively. (**C**) Footprint (see Section 2.9 for color coding information). The NT_sami_ group average values of the print length (PL), toe spread (TS), and intermediate toe spread (IT) were similar to those of the naïve group (**left** 2 panels) and significantly better relative to the SCI_mi_ controls (*, *p* < 0.05; one-way ANOVA with post hoc Tukey’s test). In the NT_dmi_ group, there were significant bilateral chronic PL deficits compared to the naïve control and NT_sami_ groups, while the SCI_mi_ rats showed marked abnormality in all three parameters (**right** two panels). (**D**) Nociception/pain reflex and (**E**) Pressure reflex. The recovery course (i.e., the time required to restore an average score of ~2) was 3 weeks and 2 weeks post NT in the NT_sami_ group for pain- (**D**) or pressure-triggered (**E**) hindlimb withdrawal reflex, respectively. NT significantly improved the two reflexes relative to SCI_mi_ controls (*, *p* < 0.05; two-way repeated measures ANOVA with Tukey’s post hoc test). However, NT_dmi_ did not generate significant benefits for the two sensory functions compared to the control group (**D**,**E**).

The naïve and LAM/NT_sham_ groups did not show any detectable behavioral abnormalities. In contrast, all SCI animals after mild or moderate thoracolumbar contusion exhibited a more serious loss of hindlimb function at 1 day p.i., with a group average BBB score of 7.5 and 6, which indicated extensive movements of three or two hindlimb joints without bodyweight support, respectively. These scores suggested that neither T13-L1 thoracolumbar contusion type resulted in spinal shock (i.e., transient loss of spinal cord function caudal to the level of the injury: flaccid paralysis, anesthesia, absent bowel and bladder control, and areflexia) that occurs acutely after the same weight-drop insults to the lower thoracic cord (e.g., T8 and T9-10) [27,45]. Afterward, very limited functional amelioration arose in SCI_mi_ rats (i.e., ~3 BBB scores), plateauing (i.e., a mean BBB score of ~10.5; Figure 2A(i)) by 9 weeks p.i. to represent the long-term deficits characteristic of mild T13-L1 contusion. SCI_mo_ animals showed a weaker spontaneous recovery (i.e., ~2.5 BBB scores), with a flat score curve between week 2 and week 6 p.i. By 7 weeks p.i., the group average BBB score again dropped 1–1.5 BBB scores (Figure 2A(ii)). In this study, all included SCI animals receiving NT treatment demonstrated certain degrees of BBB improvement (Figure 2A; see Section 2.9 for study inclusion specifics).

The injury control animals showed a similar pattern of impairment in their incline plane performance. When positioned facing downward, the group mean angle of the SCI_mi_ and SCI_mo_ rats was 34° and 35° at 1 day p.i., respectively, indicating no presence of spinal shock. These angles had limited improvements (i.e., ~10°) before reaching a plateau of 45° and 42° by 2 weeks p.i., representing long-term deficits typical for T13-L1 mild or moderate contusion, respectively (Figure 2B). Similar to lower thoracic SCI [27,45], when forelimb strength (i.e., general physical condition) was examined in the upward-facing orientation, no deficits were observed in either group.

### 3.2. T12-L3 NT-Mediated Recovery of Coordinated Hindlimb Functions after T13-L1 Contusion

Locomotor performance. To test whether T12-L3 NT might induce hindlimb improvement after thoracolumbar contusion, coordinated hindlimb function and evolution of hypersensitivity/pain were evaluated as clinically relevant parameters [26]. In addition, the pressure-triggered withdrawal reflex was monitored to evaluate deep touch pressure sensation that comprises tactile and proprioceptive inputs, since proprioception is a main mechanism underpinning post-SCI locomotion [20,54]. For rats with unilateral NT, the hindlimb ambulating ability and stability on the incline plate showed bilateral improvement, with the non-NT hindlimb having a slightly weaker performance (i.e., −1 to −2 BBB scores). According to an established rule in SCI therapeutic research [29], outcomes of the better side from applicable behavioral tests were used to present the level of recovery for a particular animal in either the unilateral or bilateral NT group to minimize chances of underrating the potential therapeutic effect.

By 2 weeks after NT (i.e., 3 weeks p.i.), NT_sami_ rats began having noticeable locomotion enhancement, and in week 4 p.i., they displayed bodyweight-bearing hindlimb stepping that ipsilaterally coordinated with the forelimbs (i.e., a group mean BBB score of ≥12). This group reached an average BBB score of 13–14 between 5 and 10 weeks and 15–16 by 11 and 12 weeks p.i. (Figure 2A(i)). Of note, the score of 12 represents a threshold of a qualitative BBB scale upgrading conversion (i.e., from non-coordinated weightbearing stepping to occasional coordinated ones) [43]. NT_samo_ animals showed a similar trend of locomotor recovery, with the group average BBB score climbing to ~12 in week 4 p.i. and ~13.5 in 7 to 12 weeks p.i. (Figure 2A(ii)). Either group had significantly better hindlimb locomotion than its control (SCI_mi_ or SCI_mo_) group (*p* < 0.05; two-way repeated measures ANOVA with Tukey’s post hoc test). Delayed NT and i.m. FGF2 release, given 8 weeks after the SCI and 1 week post near-epicenter hMSC injection (plus EEE housing), also significantly benefited locomotor function of NT_dmi_ and NT_dmo_ groups relative to each’s control group (Figure 2A(i,ii); *p* < 0.05). However, it did not induce BBB scale upgrading conversion in NT_dmo_ animals (i.e., group average BBB scores: <12; Figure 2A(ii)).

Incline plane. The group average angles in the upward-facing orientation, which reveals forelimb strength that is not disturbed by a T13-L1 contusion, were similar between the groups. In contrast, performance facing downward as a measure of coordinated hindlimb function detected significant improvements in NT_sami_ and NT_samo_ groups compared to corresponding controls (*p* < 0.05; two-way repeated measures ANOVA with Tukey’s post hoc test; Figure 2B). Specifically, NT_sami_ animals showed a significantly increased group mean angle starting two weeks after NT (week 3 p.i.: 47°), with continuous improvement until week 7 p.i. (54°), where the performance stabilized until the end of the study (compared to 45° of SCI_mi_ in weeks 7–12). NT_samo_ animals improved the average angle from 47° (2 weeks post NT/3–4 weeks p.i.) to 50° (8 weeks p.i.) and plateaued thereafter (Figure 2B; versus SCI_mo_’s ~43° in weeks 8–12). In a lesser scale, the group mean angle of NT_dmi_ and NT_dmo_ rats was significantly improved starting 2 weeks (10 weeks p.i.: 52°) and 3 weeks (11 weeks p.i.: 48°) after neurotization, respectively (Figure 2B).

Footprint. The NT_sami_ and NT_dmi_ animals were examined for more detailed functional impact of the T12-L3 neurotization through footprint analysis. The walking track of the hindpaw with the NT-treatment was measured to generate parameters that were compared to those of SCI_mi_ animals. The NT_sami_ group average values of all three outcome measurements, i.e., the print length (PL), toe spread (TS), and intermediate toe spread (IT), were significantly improved in the NT-treated hindpaws relative to the controls (*p* < 0.05; one-way ANOVA with Tukey’s post hoc test; Figure 2C). Furthermore, the mean values of PL, TS, and IT of the NT-treated hindpaw were statistically indiscernible from those of the naïve control group or the non-NT side footprints, which corroborated the locomotion data (i.e., unilateral T12-L3 NT resulted in bilateral hindlimb functional benefits in NT_sami_ rats) (left two panels of Figure 2C). Lastly, in the NT_dmi_ group, there were significant bilateral chronic PL deficits compared to the naïve control and NT_sami_, and the SCI_mi_ rats showed marked abnormality in all three parameters (right two panels of Figure 2C).

### 3.3. T12-L3 NT-Induced Sensory Reflex Improvement

As per the same arrangement of the footprint assay, sensory alterations were evaluated by testing spinal neurological reflexes for the NT_sami_, NT_dmi_, and SCI_mi_ groups only to minimize physical disturbance to rats with moderate SCI. These outcomes were presented in the mean score ± S.E. of each group. Overall, spinal withdrawal reflexes in reduced amplitudes (i.e., hyporeflexia) triggered by sharp-tip pinching (nociception/pain reflex) and brief blunt pinning (pressure reflex) of the hindpaw were detectable in most SCI_mi_ rats at 1 day p.i., which was consistent with the locomotor and incline plane data (see above), reconfirming no occurrence of spinal shock at 1 day after mild thoracolumbar contusion (Figure 2D,E). The recovery course (i.e., the time required to reach and stabilize around an average score close to 2, which defines a state of normoreflexia or minor hyporeflexia) was 3 weeks (4 weeks p.i.) and 2 weeks (3 weeks p.i.) in the NT_sami_ group for pain- (Figure 2D) or pressure-triggered (Figure 2E) withdrawal reflex, respectively. The results were significantly better than those of SCI_mi_ controls that developed hyperreflexia to nociceptive input but remained hyporeflexive in response to pinning/pressure (*p* < 0.05; two-way repeated-measures ANOVA with Tukey’s post hoc test). Conversely, NT_dmi_ did not generate significant benefits for the two sensory functions, compared to the control group (Figure 2D,E).

### 3.4. The Impact of T12 Dorsal Root Input on the T12-L3 NT-Facilitated Locomotion

**Figure 3:**
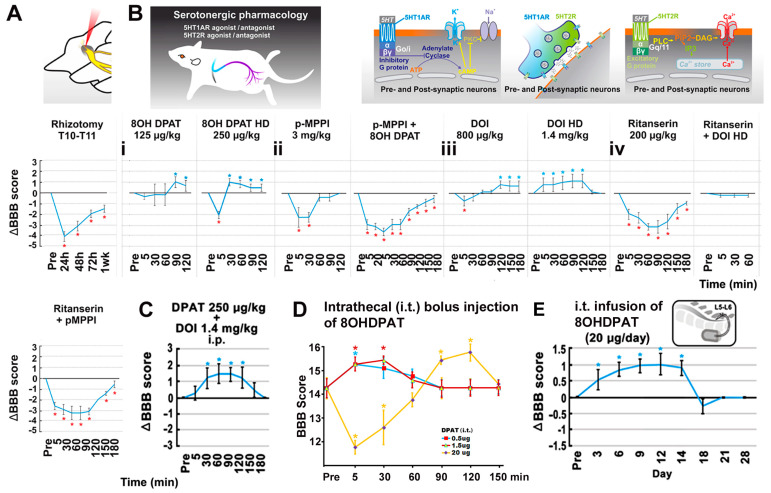
Data of rhizotomy and pharmacological neuromodulation. (**A**) Dorsal rhizotomy. On average, the surgical ablation of the T12 dorsal root reduced the mean BBB score by 4 points in the ipsilateral hindlimb at 1 day post rhizotomy, which remained significantly below the pre-rhizotomy BBB level for 7 days (*, *p* < 0.05; one-way ANOVA with Tukey’s post hoc test; n = 5). (**B**–**E**) Serotonergic pharmacology. (**B**(**i**)) Bolus 5HT1A receptor agonist 8OHDPAT (n = 5/NT_sami_ rats) dose-dependently augmented BBB scores for ~90 min (*, *p* < 0.05; two-way ANOVA with Tukey’s post hoc test). The effect was blocked by pre-dosing of p-MPPI, a 5HT1A-specific antagonist (**B**(**ii**)). (**B**(**iii**)) Bolus DOI administration, a preferential 5HT2A receptor agonist, dose-dependently increased the average BBB score in NT_sami_ animals, with the lower dose exerting a short initial inhibition before eliciting enhancement, and the higher dose having an exclusive positive effect (*, *p* < 0.05; one-way ANOVA with Tukey’s post hoc test; n = 5). (**B**(**iv**): first plot) Injection of 5HT2A antagonist ritanserin (n = 5) inhibited baseline BBB scores for ~180 min. (**B**(**iv**): second plot) Pre-administration of ritanserin blocked the locomotor stimulation effect of DOI (n = 5); however, the combinatorial application of ritanserin (200 µg/kg, i.p.) and p-MPPI (3 mg/kg, i.p.) suppressed the BBB score for 180 min (n = 5; (**B**(**iv**): third plot). (**C**) Co-administration (i.p.) of 8OHDAP (250 µg/kg) and DOI (1.4 mg/kg) in NT_dmi_ rats produced mild additive effects (*, *p* < 0.05; n = 5) for 120 min. (**D**) Bolus intrathecal (i.t.) doses of 8OHDPAT significantly increased BBB scores for ≥30 min, with 20 µg 8OHDPAT showing an initial inhibition followed by a stimulating effect. (**E**) Lastly, chronic i.t. infusion of 8OHDPAT (20 µg/12 µL/day × 14 via an s.c. implanted Alzet^®^ osmatic minipump of 14 days capacity) to NT_samo_ animals significantly enhanced the mean BBB score for ≥11 days (*, *p* < 0.05; two-way repeated-measures ANOVA with Tukey’s post hoc test; n = 5).

Our previous study demonstrated that T12-L3 NT performed within a time window that was supportive for sensory nerve reinnervation is key to locomotor enhancement after T13-L1 hemisection [19]. The role of the T12 dorsal root afferent input versus that of T12 ventral root in mediating T12-L3-induced hindlimb recovery was first analyzed through T12 dorsal rhizotomy (Figure 3A) in NT_sami_ rats with unilateral neurotization in week 16 p.i. (i.e., 4 weeks after BBB scoring; n = 4). On average, the surgical ablation of the T12 dorsal root reduced the mean BBB score (pre-rhizotomy score: ~15.7) by 4 points to a score of ~11 (i.e., <12 and no significant difference than the SCI_mi_ group) in the ipsilateral hindlimb at 1 day post rhizotomy, which remained significantly below the pre-rhizotomy BBB level for 7 days before gradually returning to the pre-surgery level (Figure 3A). In contrast, T12 ventral root rhizotomy performed for NT_samo_ animals with unilateral anastomosis within the same timeframe did not significantly reduce the pre-rhizotomy mean BBB score (i.e., ~12.5 ± 1.22 versus ~13.9 ± 1.18; n = 4). The data suggested that the T12 dorsal root more pivotally contributed to post-NT locomotor recovery; however, at 16 weeks p.i. and ~15 weeks after T12-L3 neurotization, other NT-ignited neuroplastic mechanisms might eventually compensate for the T12 dorsal root afferent deficit.

### 3.5. The Effect of 5-HT Neuromodulation on the T12-L3 NT-Mediated Locomotor Recovery

The contribution of intraspinal 5HT neuromodulation (normally via RpST) [55] to hindlimb improvement resulting from T12-L3 NT treatment was pharmacologically examined in NT_sami_ rats with bilateral neurotizations. The bolus systemic administration of selective 5HT1A receptor agonist 8OHDPAT (125 or 250 µg/kg, i.p. at 12 weeks p.i.; n = 5/NT_sami_ rats) dose-dependently enhanced locomotor performance for ~90 min. The higher dose produced a transient (peaked at 5 min after drug injection) depression of locomotor function (i.e., −2 BBB scores) (*p* < 0.05; two-way ANOVA with Tukey’s post hoc test; Figure 3B(i)). The effect appeared to be mediated via activating 5HT1A receptors because the pre-dosing of p-MPPI (3 mg/kg, i.p.), a 5HT1A specific antagonist, reversed the effect of 8OHDPAT (Figure 3B(ii)). Notably, injection of the same dose of p-MPPI alone caused a significant reduction in the baseline BBB score for ~30 min (see Section 4 for possible mechanisms involved).

Next examined was the effect of the preferential 5HT2A receptor agonist DOI (with weak affinity to 5HT2C receptors) [56] on the NT-improved locomotion [57]. Bolus DOI administration (800 µg/kg and 1.4 mg/kg; i.p. at ≥12 weeks p.i.) dose-dependently increased the group average BBB score in NT_sami_ animals; the lower dose had a short initial inhibitory impact (~5 min) before eliciting a detectable enhancement (↑0.5 point of the mean BBB score), and the higher dose showed an exclusive positive effect (↑the mean BBB score by 1 point; *p* < 0.05; one-way ANOVA with Tukey’s post hoc test; n = 5; Figure 3B(iii)). The injection of ritanserin (200 µg/kg, i.p.; n = 5), a 5HT2A antagonist that has a slightly weaker affinity to 5HT2C receptors [58], inhibited group average BBB scores by a range of 1–3 scores for 180 min; however, unlike p-MPPI (see above), pre-administration of ritanserin in the same dose only blocked the locomotor stimulation effect of DOI (n = 5; Figure 3B(iv)).

The combinatorial i.p. application of ritanserin (200 µg/kg) and p-MPPI (3 mg/kg) suppressed the baseline BBB score by 0.5–3 points for 180 min (n = 5; Figure 3B(iv)), suggesting that T12-L3 NT might have augmented 5HT availability to activate 5HT1A and 2A receptors in the lumbosacral region through increasing 5HT neurite regrowth to improve locomotion [20,25,59,60]. Indeed, co-administration (i.p.) of 8OHDAP (250 µg/kg) and DOI (1.4 mg/kg) in NT_dmi_ rats only showed mild additive effects (↑1.5 BBB score for 120 min; *p* < 0.05; n = 5), indicating a limited impact of bolus delivery of 5HT1A and 2A agonists (Figure 3C). To avoid the deleterious systemic side effects of 5HT receptor agonists (e.g., the psychedelic impact of 5HT2A agonists) [61], the local bolus effect of smaller doses of 8OHDPAT on the spinal cord was evaluated. Both 0.5 µg and 1.5 µg bolus doses of 8OHDPAT (i.t.) significantly enhanced post-NT locomotor function for ≥30 min, and 20 µg i.t. injection of 8OHDPAT showed an initial reduction of ~3 BBB points for ~30 min followed with an improvement of ~2 BBB points for ~30 min (Figure 3D). The outcome suggested that a continuous i.t. release of a 5HT1A agonist without drastic dose fluctuation might provide a steady boost to locomotor recovery. This hypothesis was tested in a study of chronic i.t. infusion of 8OHDPAT (20 µg/12 µL/day × 14 via an s.c. implanted Alzet^®^ osmatic minipump of 14 days capacity) to NT_samo_ animals 12 weeks after bilateral NT. The treatment significantly augmented the mean BBB score starting from day 3 of the drug infusion without triggering observable adverse behaviors. The effect lasted for ≥11 days (i.e., 14 days post pump installation) before winding down gradually over 4 days (*p* < 0.05; two-way repeated-measures ANOVA with Tukey’s post hoc test; n = 5; Figure 3E), confirming that the efficacy was produced by the effect of i.t. 8OHDPAT. These results collectively demonstrated that the serotonergic neuromodulation through 5HT1A and 2A receptors was impactful in stimulating the T12-L3 NT-modified intraspinal neurocircuit and neurotransmission to promote hindlimb function following thoracolumbar SCI.

### 3.6. Electrophysiological Evaluation of the Effect of T12-L3 NT on the Neurocircuit Integrity

**Figure 4:**
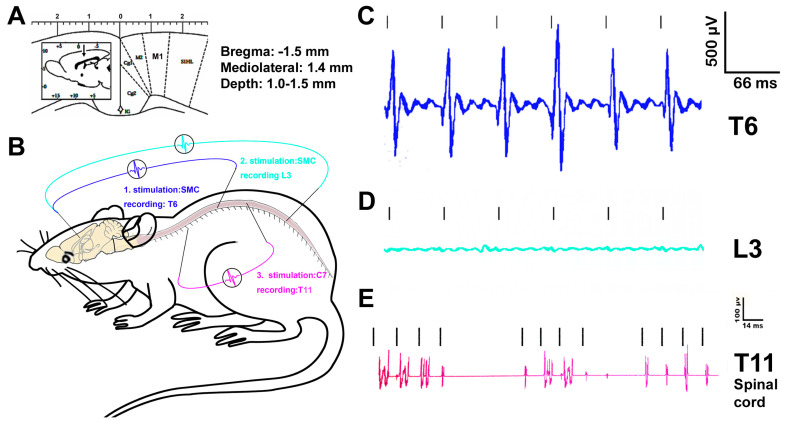
Data of electrophysiology. The drastic difference between the representative signal amplitudes of the motor cortex stimulation-evoked spinal cord potentials (MESPs) recorded from T6 (above injury level) and those at the L3 spinal cord (below injury) confirmed that T13-L1 contusion disrupted the corticospinal tract in animals with NT_sami_ at 14 weeks p.i. (n = 4; (**A**–**D**)). Spinal cord stimulation-evoked potentials (SEPs) were readily detectable from C7 dorsal root stimulation for recording from T11 IMG in NT_sami_ animals (n = 4; (**E**)) but not in the SCI controls (n = 3), suggesting that the NT_sami_ treatment might have strengthened the functional integrity and capacity of the intraspinal neurocircuitry.

**Figure 5:**
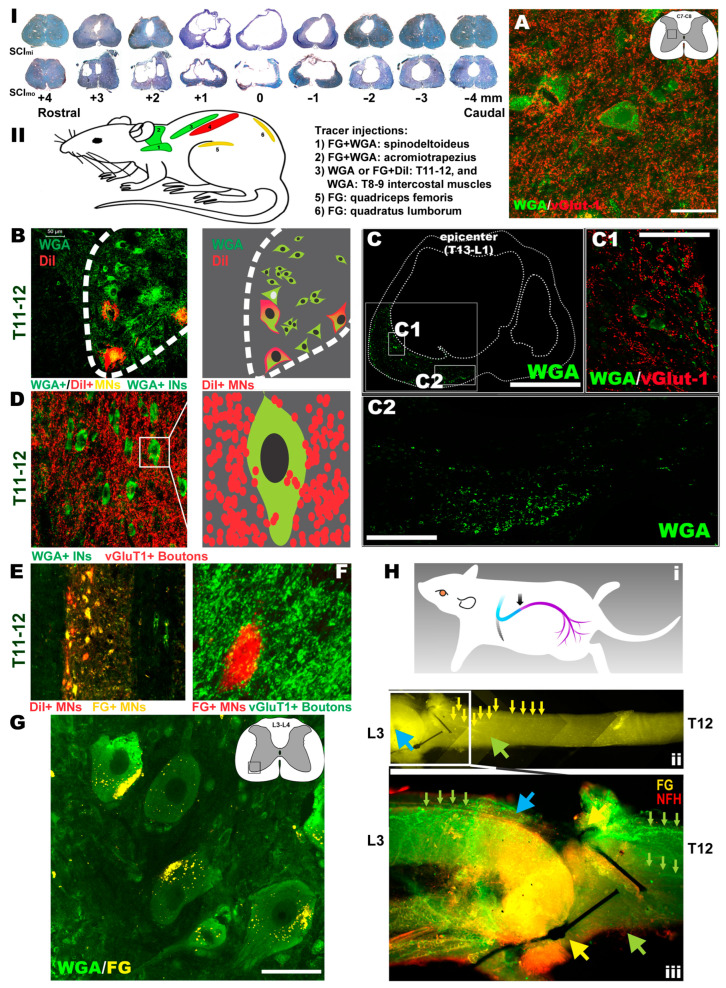
Outcomes of histopathology, neural tracing, and immunohistochemistry. There were apparent lesions in white matter (WM) and gray matter (GM) at the contusion epicenter, with cavities and lesions extending rostrally and caudally to demarcate the lesion volume and the epicenter coronal pathological profile of the mild and moderate contusion injuries ((**I**); note: quantified assays did not find any overall significant statistical differences of tissue sparing between the groups with either SCI_mi_ or SCI_mo_, respectively). (**II**) A neural/neuromuscular tracing revealed that in the NT_sami_ animals, WGA rostrocaudally traced C7-8 INs in the IMG (inset in the schematic) that were surrounded by vGlut1+ neurite terminals (bar: 40 µm; (**A**)). Also labeled were T11-12 MNs (WGA+/DiI+) and INs (WGA+) (**B**), with WGA being relayed by the IN axons ((**C**); bar: 1 mm) and INs spared at the epicenter. These INs received vGlut1+ proprioceptive terminals (**C**(**1**); bar: 80 µm). Many of the neurites (WGA+/green) passed through the residual parenchyma (**C**(**2**); bar: 200 µm) before reaching lumbar neurons. Some of the tracer+ projection neurites were from the cervical and thoracic propriospinal INs ((**A**,**D**), respectively) as they were WGA+ and encircled by vGlut1+ neurite terminals. The T11-12 VH MNs (FG+/DiI+; (**E**)) received dense contacts of WGA+ proprioceptive fibers ((**F**): FG shown in red), and the WGA tracers trans-synaptically reached lumbar MNs (bar: 50 µm; (**G**)). FG retrograde tracing (**H**(**i**)) entered the T12-L3 nerve (**H**(**ii**)), with FG particles coming across the anastomosis site and the T12 nerve to move toward the spinal cord (blue and green arrowheads and small yellow arrows in (**H**(**ii**)). The FG fluorescent signal (yellow) overlapped with that of neurofilament-H (NF-H: red), showing green-colored (yellow + red) axon bundles (blue arrowhead and small green arrows in (**H**(**iii**)). Some FG tracers via L3 and other lumbar nerves also entered the lumbar VH MNs (yellow; (**G**)). Conversely, DiI traced the T11-12 MNs (red, (**E**)) that innervated the intercostal muscles (see tracer injection sites in (**II**)).

Motor-evoked spinal cord potentials. The typical signal amplitudes of the motor cortex stimulation-evoked spinal cord potentials (MESPs) recorded from T6 (above injury level) versus those at L3 spinal cord (below injury) were presented to qualitatively show the effect of T13-L1 contusion on the CST function in animals with NT_sami_ at 14 weeks p.i. (Figure 4A–D). While the MESPs were strongly detectable at T6 (Figure 4C), they were absent in the L3 spinal cord of either NT_sami_ (n = 4; Figure 4D) and SCI_mi_ animals (n = 3), proving that the mild contusion blocked the CST conduction at the epicenter (Figure 5A) and T12-L3 NT did not induce CST regeneration.

Spinal cord stimulation-evoked potentials (SEPs). To assess the functional integrity of the intraspinal cord neurocircuit (between C7 and T11) containing the cervical long descending and thoracic propriospinal projection and descending 5HT pathways [50,62], the characteristic signals of SEPs, which were evoked from C7 dorsal root stimulation and recorded at T11 IMG, were collected (Figure 4B). The SEPs were clearly detectable in the NT_sami_ animals (n = 4; Figure 4E). In contrast, such SEPs were negligible in the SCI_mi_ controls (n = 3; data not shown). Thereby, the NT_sami_ treatment might have promoted pro-repair neuroplasticity partially via enhancing the functional integrity and capacity of the intraspinal neurocircuitry, including the propriospinal projection network (see details in the neural tracing data below). It is also important to note that whether similar levels of spinal cord potentials could be evoked in NT_samo_ rats remains to be investigated.

### 3.7. Histopathological Outcomes

Representative spinal cords in the SCI_mi_ and SCI_mo_ groups exhibited apparent lesions in the white matter (WM) and gray matter (GM) at the contusion epicenter, with cavities and tissue lesions extending rostrally and caudally to demarcate the lesion volume as well as epicenter coronal pathological profile of the mild and moderate thoracolumbar contusion injuries (Figure 5I). Digital microscopic images of archetypal cross sections of the lesion volume were quantified for the amount of residual tissues. There were no overall significant statistical differences of WM or GM sparing between the groups with either SCI_mi_ (i.e., NT_sami_, NT_dmi_, and SCI_mi_) or SCI_mo_ (i.e., NT_samo_, NT_dmo_, and SCI_mo_), respectively (*p >* 0.05; two-way ANOVA; n = 7 representative animals/group). Therefore, the general histopathological damages after T13-L1 mild or moderate contusion were not discernibly ameliorated by either subacute (7–9 days p.i.) or delayed (8 weeks p.i.) NT intervention.

### 3.8. Neural and Neuromuscular Tracing and IHC Analysis

**Figure 6:**
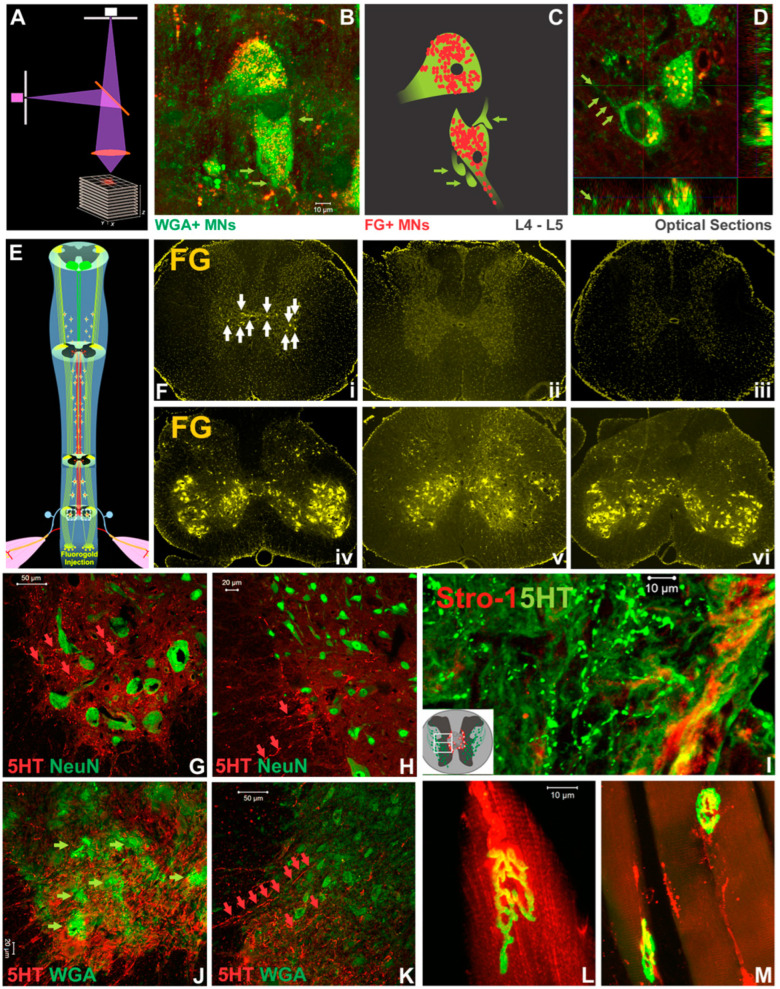
Results of neurocircuit and NMJ analyses. Confocal z-stack imaging of the tissue (schematics in (**A**) revealed that QF-originated FG and ACZ/SPD-derived WGA both traced L4-5 VH MNs (**B**–**D**). Notably, in NT_sami_ animals, WGA+ MNs were thickly surrounded by WGA+ proprioceptive neurite terminals and presynaptic boutons (green arrows in (**B**), (**C**) digital schematics, and (**D**) optical slicing). Thoracic propriospinal projection was enhanced by the NT as confirmed by intra-L3-4 spinal cord FG tracer (**E**) that was picked up by T10 INs in NT_dmi_ cords (**F**(**i**): white arrows placed toward T10 INs; **F**(**iv**): a L4 section), and not in NT_dmo_ (**F**(**ii**,**v**)) or SCI_mi_ (**F**(**iii**,**vi**)) tissues. To evaluate below-injury level 5HT reinnervation, confocal images of IHC co-staining of 5HT and other markers showed that representative NT_sami_, not injury control, tissue sections had ample 5HT+ axons (red arrows in (**G**,**H**)) extending toward NeuN+ L3 MNs (**G**) and INs (**H**). These 5HT+ neurites coursed through epicenter residual parenchyma as evidenced by their presence above the lesion site in NT_dmi_ tissues where Strol-1, an hMSC marker, was also detected (**I**). In NT_sami_-treated cords, these 5HT+ fibers tracked WGA+ VH MNs ((**J**): green arrows) and IMG INs ((**K**): 5HT fibers are indicated by red arrows) in L3 region. In NT_sami_ animals that had DiI and FG administered to QF and QL, the morphological integrity of NMJs was preserved ((**L**) and (**M**), respectively: uptake of DiI or FG by the NMJ which was outlined via α-bungarotoxin IHC in green).

Neural and neuromuscular tracing. To test the hypothesis that if compared to the SCI_mi_ and NT_dmi_ animals, neural tracing in the NT_sami_ group, which demonstrated the largest average scale of hindlimb recovery, might have the best opportunity to reveal key neuropathway mechanisms underlying the NT-improved locomotion, typical NT_sami_ and NT_dmi_ animals with unilateral neurotization as well as SCI_mi_ controls (n = 4–5/each) were included for the neural and neuromuscular tracing assays (Table 3). Following the tracing design (note: all tracers were injected 4–8 weeks before animal termination), FG (a retrograde tracer) and WGA (a trans-synaptic tracer) were administered into the acromiotrapezius (ACZ) and spinodeltoideus (SPD; innervated by C2-C7 nerves) [63,64]. WGA or DiI (a bidirectional tracer) were introduced into the T8-9 (WGA) and T11-12 (DiI) intercostal muscles (innervated by lower thoracic spinal nerves) [65]. FG was administered into the quadriceps femoris (QF) or quadratus lumborum (QL) of NT_sami_ animals, primary muscles innervated by the lumbar nerves [35], including the T12-L3-neurotized nerve (Figure 5II). In the NT_sami_ animals, WGA rostrocaudally traced C7-8 INs in the IMG that were surrounded by vGlut1+ neurite terminals (i.e., presynaptic boutons), indicating that they were members of the propriospinal projection network (Figure 5A) [66,67]. T11-12 MNs (WGA+/DiI+) and INs (WGA+) were strongly labeled, with WGA being relayed by the axons (Figure 5C) to the epicenter surviving INs receiving vGlut1+ proprioceptive terminals (Figure 5C(1)), or directly through the residual T13-L1 parenchyma (Figure 5C(2)) to reach lumbar neurons. Some of these projection neurites might have originated from the cervical and thoracic propriospinal INs (Figure 5A,D, respectively) as they were WGA+ and encircled by vGlut1+ neurite terminals. In addition, the T11-12 VH MNs (FG+/DiI+; Figure 5E) received dense contacts of WGA+ proprioceptive fibers (Figure 5F), and the WGA tracer was trans-synaptically transported into lumbar MNs (Figure 5G; more details in Figure 6). The results suggested that there was augmented neuroplastic activities in the lower cervical and thoracic propriospinal projection systems induced by the T12-L3 NT, since such tracer signals were much weaker in the NT_dmi_ animals or not seen in the SCI_mi_ controls.

Caudorostrally, FG retrograde tracing (Figure 5H(i)) was found inside the T12-L3 nerve (Figure 5H(ii)), with FG particles traveling across the anastomosis site entering the T12 nerve to move toward the spinal cord (Figure 5H(ii)). Coursing through the T12 nerve, the FG fluorescent signal (yellow) overlapped with that of neurofilament-H (NF-H: red), showing green-colored (yellow + red) axon bundles (blue arrowhead and small green arrows in Figure 5H(iii)). Some FG particles transported by L3 and other lumbar nerves also entered the lumbar VH MNs (yellow; Figure 5G; confocal imaging data in Figure 6). The data suggested that the T12 axons had regenerated through the L3 root and partially innervated the QF/QL of that animal. In contrast, DiI presented inside the T11-12 effector MNs (red, Figure 5E), illuminating intercostal muscle innervation from these neurons.

Confocal z-stack imaging analysis of tracing and IHC data (Figure 6A) revealed that QF-originated FG and ACZ/SPD-derived WGA both traced L4-5 VH MNs of NT_sami_ animals (i.e., the presence of intra-MN FG and peri-/intra-MN WGA signals; Figure 6B–D). Of note, these WGA+ MNs were thickly surrounded by WGA+ proprioceptive Ia appearing terminals and presynaptic boutons (Figure 6B; digital schematics in Figure 6C; optical slicing in Figure 6D), indicating the improved cervical propriospinal neurite projections. Thoracic propriospinal project was also enhanced by the NT: intra-L3-4 spinal cord FG tracer (Figure 6E,F(iv)) was picked up by T10 INs in NT_dmi_ animals (Figure 6F(i)), not by NT_dmo_ (Figure 6F(ii,v)) or SCI_mi_ (Figure 6F(iii,vi)) controls. Finally, BDA tracing did not detect post-NT CST regrowth in the injured cords.

To evaluate the effect of T12-L3 NT on below-injury serotonergic reinnervation, confocal images of IHC co-staining of 5HT and other markers were analyzed. Representative NT_sami_, not injury control, spinal cord sections had abundant 5HT+ axons that extended toward NeuN+ L3 MNs (Figure 6G) and INs (Figure 6H). These 5HT+ neurites traveled through epicenter residual parenchyma as suggested by their presence above the lesion site in NT_dmi_ tissues where Strol-1, an hMSC marker, was also found (Figure 6I). In NT_sami_-treated cords, these 5HT+ fibers tracked WGA+ VH MNs (Figure 6J) and IMG INs (Figure 6K) in the L3 region. Lastly, in NT_sami_ animals that received DiI and FG via injections to QF and QL, the morphologic integrity of NMJs was preserved (i.e., the uptake of DiI or FG by NMJs that were also labeled by α-bungarotoxin; Figure 6L and Figure 6M, respectively). Such an observation, which was absent from the SCI control tissue, is consistent with our earlier finding [19].

## 4. Discussion

Statistically significant and neurologically meaningful hindlimb recoveries post mild T13-L1 thoracolumbar contusions (SCI_mi_) were recorded following subacute (7–9 days p.i.) or delayed (8 weeks p.i.) rerouting of the T12 intercostal nerve to the distal L3 nerve root. In animals with moderate T13-L1 contusion (SCI_mo_), NT_samo_ and NT_dmo_ also produced statistically significant hindlimb improvement. However, the NT_dmo_ group did not exhibit neurological scale upgrading conversion (i.e., mean BBB score ≤12) despite receiving hMSC and FGF2 injections plus EEE housing. Conversely, hindlimb deficits in the SCI controls with sham procedures stayed permanent. The enhanced coordinated hindlimb functions and spinal reflexes after NT_sami_ intervention coexisted with T12 neurite regrowth into the L3 nerve to reach target muscles and augmented T12-L3 sensory input-related neuroplasticity. The NT-induced benefits included markedly strengthened trans-synaptic tracer (WGA) relay from cervicothoracic IMG INs to lumbar VH MNs and IMG INs through vGluT1+ propriospinal neurites (i.e., ↑functional capacity of the propriospinal network), increased reinnervation of 5HT+ RpST to below-lesion spinal cord, and maintenance of the NMJ integrity in the target muscles. Overall, the data demonstrates that T12-L3 NT induced intraspinal neuroplasticity without CST regrowth to benefit locomotion recovery, in which the neurotized sensory nerve may play a more crucial role than the ventral motor root. The results corroborate our previous data that T12-L3 NT, when performed within a time window (~1 week p.i.) in favor of sensory nerve regrowth [68,69], reanimated the paralyzed hindlimb in a rat model of T13-L1 hemisection [19]. To the best of our knowledge, this is the first study where the effects of the sensory input, propriospinal network function, 5HT neuromodulation, and NMJ integrity on mediating hindlimb locomotor recovery following mixed nerve neurotization have been systematically investigated in an in vivo model of thoracolumbar contusion (i.e., one of the most common injuries encountered by humans) [1]. 

Neurotizations utilizing clinically defined motor nerves have been applied to restore neurological functions of the upper limbs or extremities of people after PN injuries, with noticeable levels of success [12,13,14]. However, much less explored is to transfer motor and sensory mixed nerves to overcome lower limb locomotor deficits following SCI. Although some cases have shown various degrees of therapeutic potentials [70,71], the inconsistent therapeutic efficacy and neurological complications (e.g., improper motor pattern) are uncomplimentary factors that have hindered a broader acceptance and application of NT for lower limb locomotor repair after SCI. Furthermore, the conclusion that total motor reinnervation of the target muscle can be achieved by intercostal NT has been disputed [15,16,19]. Small-caliber nerves (e.g., intercostal and brachial) were deemed insufficient to account for the motor enhancement of large limb muscles [72,73]. The challenges appear to stem principally from an inadequate understanding of essential neurobiological mechanisms for functional changes induced by NT treatment. Thus, pro-repair contributions from sensory input and central neuroplasticity were hypothesized and initially tested by us in a rat model of T13-L1 hemisection [19].

The post-SCI CNS neuroplasticity process, which comprises both maladaptive and pro-repair adjustment endeavors, has been differentially targeted to optimize functional recovery due to its varied susceptibility in different p.i. stages to therapeutic interventions [74,75]. Therapeutic strategies such as epidural or transcutaneous electrical stimulation (EES or TES) were able to reactivate the central pattern generator (CPG) to restore locomotion for chronic experimental and clinical SCI [76,77]. Since conventionally defined complete injuries often spare the CPG network that resides in the lumbosacral cord [78], through delivering formulated electrical signals, EES/TES can induce muscle contraction and generate alternated lower limb movement to enable AIS-A participants to walk or cycle with certain assistance [79,80]. Therefore, the segmental spinal neural circuits, despite not directly mediating volitional actions, can elicit rhythmic motor and locomotor patterns, providing a neural network matrix for developing functional stimulation, pharmacological neuromodulation, computer interfacing, and activity-based rehabilitation [3,81,82,83]. Our published and current data have also demonstrated that the propriospinal projection, the 5HT RpST, and NMJ systems (i.e., the key components of the recovery neurobiology) [7] may be surgically manipulated through mixed NT to reanimate the CPG for hindlimb locomotor recovery after incomplete SCI [7,19,20].

The importance of somatosensory feedback in restoring locomotion post SCI that did not destroy the CPG has been exemplified in laboratory studies where tactile stimulation of the distal limbs, via applying electrical stimulation or direct mechanical disturbance, produced greater locomotion restoration [84]. Prior investigations found that in spinal cats that had hindlimb deafferentation-caused paralysis, locomotion recovery was facilitated if the L6 dorsal root was spared [42]. In cats with post-thoracic hemisection ipsilateral immobility, stepping naturally resumed without requiring CST regrowth [85]. In both cases, the sprouting of the dorsal root central branch and descending serotonergic fibers was determined as the primary compensatory mechanism [42,85]. For the hemisection model, thoracic dorsal root central sprouting also played a beneficial role (note: for either lumbar or thoracic dorsal root sprouting, no analysis was performed to specify which type of sensory enhancement was involved) [85]. Contrariwise, spinal cats showed that deafferentation of one hindlimb substantially disrupted locomotor recovery [86]. Tonic EES applied to the lumbosacral spinal cord failed to elicit rhythmic locomotion in the deafferented hindlimb of spinal rats [87]. For mice with unilateral forelimb spasm post stroke, contralateral dorsal (not ventral) root NT ameliorated the abnormality [88]. In humans, locomotor benefits were reported for operating functional electrical stimulation-driven exercise equipment and locomotor training logistics that provide somatosensory inputs [89]. It is worth noting that EES/TES-enabled locomotion restoration has been attributed to their ability to engage the spinal cord proprioceptive signal to reactivate the CPG [77,79,80].

In the present study, when tested ≥12 weeks p.i., the subacute NT-attained locomotor ability after contusion was associated with the NT-channeled reintroduction of L3 sensory signals into the thoracic (T12) spinal cord, which has strong natural plasticity because of the existence of the “crossed-intercostal” neurocircuitry [90]. Also significantly augmented was the serotonergic reinnervation of the below-injury level spinal cord. The outcomes from our multimodal assays demonstrated that subacute T12-L3 NT induced effective motor (e.g., FG back-labeling results) and sensory (e.g., WGA cross-synaptic data) reinnervation to the target muscles. The newly formed neural network was functional as the cervicothoracic level-derived WGA was trans-synaptically relayed by the intraspinal neuronal circuits (e.g., the propriospinal projection pathways as evidenced by the vGluT1 expression in the neurite terminals) to the lumbar CPG INs and effector MNs for locomotor reinstatement [7,20]. It is hereby conceivable that the NT-built neural pathway of locomotor recovery after contusive SCI partly recapitulates the developmental neurobiology in that the gaining of the initial motor ability requires peripheral afferent feedback signals in addition to descending serotonergic modulation and selective activation of the peripheral motor units before motor tasks can be executed [91]. Hypothetically, with T12-L3 NT, the afferent drive normally designated for the lumbar segment is rechanneled into the T12 level of the spinal cord to recondition a different pool of propriospinal INs for transmitting novel influence to facilitate the reorganization of the propriospinal neurocircuit to reanimate locomotion in rodents. Moreover, we earlier reported that the T12-L3 NT activated T12-region NSCs to produce BDNF [19], which may in turn induce more DRG central neurite sprouting that is a locomotion repair mechanism [42]. BDNF can also increase 5HT reinnervation [20] and protect lower thoracic propriospinal INs that are vulnerable to secondary injury insults [92] to jointly promote hindlimb neurological recovery.

In the NT_sami_ and NT_samo_ groups, anastomosis was done at 7–9 days p.i., which is a time frame that favors sensory neurite regrowth due to naturally elevated cAMP levels inside DRG neurons that peaks ~7 days after sensory neurite lesion [93]. The hindlimb functional improvement of these animals was significantly stronger than that of the NT_dmi_ and NT_dmo_ groups, for which anastomosis was performed 8 weeks p.i. when DRG neuronal cAMP already subsided. The result corroborates our prior finding that NT performed 4 weeks post T13-L1 hemisection failed to induce locomotor enhancement [19]. For chronic SCI conditions, the intraneuronal cAMP level may be boosted by the implantation of hMSCs and/or augmented physical activity of animals since hMSC and exercise-stimulated neural cells can produce BDNF that raises cAMP synthesis [94,95]. Indeed, the combinatorial regimen of hMSC and EEE housing enabled the NT_dmi_ animals to reach neurological upgrading conversion (i.e., ≥12 BBB scores). However, this regimen failed to show the same degree of efficacy in the NT_dmo_ group. The data suggested that besides the regrowth capacity of the T12 afferent neurites, the existence of an adequate quantity of spared parenchyma at the epicenter (e.g., a continuous rim of tissue in SCI_mi_ cords versus a broken thinner tissue circumference of the SCI_mo_ group), to allow passing of the propriospinal projection and serotonergic axons, may too be a critical factor in locomotor recovery. Supporting this notion is the observation that severance of the contralateral cord abolished the NT-mediated hindlimb ambulation after T13-L1 hemisection [19].

Published work has shown that rat femoral nerve motor axons specifically reinnervate a terminal nerve branch to muscle, not skin, a sensory target. This phenomenon has been termed preferential motor reinnervation [96,97]. In addition, the accuracy of muscle-oriented sensory neurite regeneration in the same nerve exhibited spontaneous correlation with the precision of motor axon regeneration [96,98]. Interestingly, some studies have reported that following mixed PN neurotization, a quicker motor than sensory axon regeneration was detected [99,100]. Therefore, it is plausible that in our study (especially for the NT_sami_ and NT_samo_ animals), the T12 motor fibers might have first arrived at L3-innervated muscles to stabilize NMJs via its trophic effect on the muscles, besides providing some limited motor output due to its smaller axon quantity compared to the original L3 nerve [101]. Reciprocally, the muscle-produced neurotrophic factors (e.g., FGF2) [102] were retrogradely transported into the spinal cord to restrengthen the newly formed T12-L3 nerve NMJs [103] and to promote 5HT axon and T12 sensory fiber regeneration to restore the function of the proprioceptor [104] to produce afferent signals and prevent spasticity onset (note: this can quickly help reanimate locomotion) [105] of the functionally denervated hindlimbs after SCI. Such propriospinal signals were subsequently conveyed downward to work in consortium with 5HT neuromodulation to reanimate the CPG for locomotor reinstallation. This may be initially investigated by tailored stimulation of the T12 dorsal root to see whether or how monosynaptic reflexes induced by Ia fiber activation (versus polysynaptic reflexes triggered by Ib input) could be recorded in the L3-innervated muscles.

In contrast to what has been demonstrated in rats, where unilateral recurrent laryngeal nerve anastomosis with the same side phrenic nerve reanimated ipsilateral diaphragm after complete C2 transection [10], the T12-L3 NT-enabled hindlimb locomotion is dependent on the T12-L3 dorsal root input and 5HT neuromodulation. This suggests that the lumbosacral CPG may be neurobiologically different from the phrenic neurocircuit, as the latter can display clinically meaningful neuroplasticity naturally or post mild stress [106,107]. Hence, future investigations should specifically determine how T12-L3 motor nerve reinnervation, L3-T12 afferent feedback, 5HT neuromodulation, and NMJ integrity may differentially and jointly improve the T12-L3 NT-induced locomotor recovery after thoracolumbar contusion [20]. The anticipated outcome will help to determine how NT-restored locomotion is different from CST-operated ambulation [7,20,42].

For this first proof-of-principle multimodal study, there were some obvious limitations. As examples, the current research scope preempted possibilities for investigating the post-SCI locomotor effect of T12 ventral root-only (mainly motor axons plus some sensory fibers) [85,108] versus that of T12 dorsal root (sensory axons)-alone neurotization. Also not tested was the impact of the temporal order of NT (e.g., providing T12-ventral-root NT first and T12-dorsal-root NT later, or vice versa) on the neurological outcome. The existence and scale of intraspinal cord collateral sprouting of T12-L3 proprioceptive neurites were not measured in comparison to those of the touch, nociception/pain, temperature, and vibration sensory fibers. No specific molecular or genetic tactics were utilized to block the propriospinal network for the purpose of assessing its role in T12-L3 NT-reanimated locomotion [109], and 3D hindlimb kinematics were not monitored to determine whether T12-L3 NT-induced hindlimb gait is systematically different from that driven by neurophysiology or other NT modalities [110,111]. The NT_samo_ and NT_dmo_ animals might have their own unique neuroplastic mechanisms that need to be examined separately. Lastly, upcoming studies should invest more in making mechanism-probing data truly quantifiable via enhancing the specificity of a particular assay (e.g., spatiotemporal transcriptome) for testing a specific neurobiological event.

In summary, our data suggest that in addition to the conventional objective of reconstructing motor nerve innervation to control denervated muscles, NT possesses desirable potential to engender new neurocircuitry and neuroplasticity to induce recovery neurobiology-based repairs (i.e., igniting propriospinal and 5HT neurotransmission, maintaining the NMJ, muscle spindle and Golgi tendon organ, and reactivating CPG) to reinstall somatomotosensory function after SCI and other types of neurological disorders. Under this novel theoretical framework and reasoning, NT can be additively or synergistically applied in combination with other promising therapeutic strategies including neuroprotection, stem cell biology, functional electrical stimulation, medical gas therapy, pharmacological neuromodulation, and neural rehabilitation, all aiming to generate beneficial activities of the NT-built and/or intrinsic developmental neurocircuits to multimodally augment the compensation capacity of the injured adult mammalian spinal cord to attain more neurological improvement, and to mitigate the occurrence of debilitating complications.

## Figures and Tables

**Table 2:** Pharmacological treatments.

Drug	Effect	Route	Dose	Vendor	References
8-OH-DPAT: 8-hydroxy-2-(di-n-propylamino)tetralin	5HT1A receptor agonist	i.p. i.t.	0.125 or 0.25 µg/kg 20 µg/day × 14	Tocris Bioscience	[25,39]
p-MPPI: 4-(2′-Methoxyphenyl)-1-[2′-[N-(2″-pyridinyl)-p-iodobenzamido]ethyl]piperazine	5HT1A receptor antagonist	i.p.	3 mg/kg	Tocris Bioscience	[25]
DOI: 1-(2,5-dimethoxy-4-iodopheny)-2-aminopropane	5HT2A receptor agonist	i.p.	0.8 or 1.4 mg/kg	Sigma-Aldrich	[40]
Ritanserin: 6-[2-[4-[Bis(4-fluorophenyl)methylene]-1-piperidinyl]ethyl]-7-methyl-5*H*-thiazolo[3,2-*a*]pyrimidin-5-one	5HT2A receptor antagonist	i.p.	1.5 mg/kg	Tocris Bioscience	[41]

Notes: (a) all i.p. injections were in 0.5 mL final volume; (b) p-MPPI: p-MPPI monohydrochloride; (c) DOI: DOI hydrochloride; (d) 8-OH-DPAT, p-MPPI, and DOI were dissolved in sterile 0.9% saline (pH adjusted to 7.4); (e) Ritanserin was dissolved in a sterile vehicle of 95% isotonic saline, 2.5% ethanol, and 2.5% cremaphor (Fluka Chemicals Ltd., Gillingham, Dorset, UK); (f) i.t.: intrathecal.

**Table 3:** Neural tracing.

Tracer	Location	Administration Side
**NT_sami_**		
2% FG	Quadriceps Femoris or Quadratus Lumborum	Unilateral (NT side; 1 mm^3^ Gelfoam × 4/muscle)
DiI crystal + 2% WGA (10 µL)	T11-T12 intercostal muscles	Unilateral (NT side)
**NT_sami_/NT_dmi_/SCI_mi_**		
10% BDA (2 µL × 10)	Primary motor cortex	Contralateral to NT side
2% WGA (10 µL)	T8-T9 intercostal muscles	Unilateral (NT side)
DiI crystal + 2% FG (10 µL)	T11-T12 intercostal muscles	Unilateral (NT side)
2% FG + 2% WGA (10 µL)	Spinodeltoideus (SPD) and acromiotrapezius (ACZ)	Unilateral (NT side)
DiI crystal + 2% WGA (10 µL)	Quadriceps Femoris	Unilateral (NT side)
2% FG (10 µL)	L3 lumbar cord	Bilateral (lateral white matter)
**NT_dmi_/NT_dmo_/SCI_mi_**		
DiI crystal + 2% WGA (10 µL)	Quadriceps Femoris	Unilateral (NT side)
2% FG (10 µL)	L3 lumbar cord	Bilateral (lateral white matter)

**Table 4:** Antibodies for IHC analysis.

Name	Host	Vender	Dilution
**Primary antibody**			
Donor hMSC:			
STRO-1	Mouse	Santa Cruz Bio.	1:400–500
Neurotransmission:			
vGluT1	Mouse	MilliporeSigma	1:200
5HT	Rabbit	Bio-Rad Laboratories	1:10,000
5HT	Rabbit	Immunostar	1:2000
Neuron:			
Neurofilament H	Rabbit	Chemicon	1:1000
Neuromuscular junction:			
α-bungarotoxin		Molecular Probes	1:200
**Secondary antibody**			
Alexa Fluor^®^ 488 Donkey		Jackson ImmunoRes.	1:400–600
Alexa Fluor^®^ 647 Donkey Anti-Rabbit IgG (H+L)			
Anti-Mouse IgG (H+L)			

## Data Availability

All data will be accessible upon written request after the scientific results are published. All experimental materials are commercially available.

## Supplementary Materials

The following are available online at https://www.mdpi.com/article/10.3390/cells12242804/s1, Figure S1: the effect of environmentally enriched/enlarged (EEE) housing on physical activity level of SCI rats. NT_dmi_ rats were housed either in traditional cages ((a), left panel: 16″ × 8″ × 8″ = 1024 in^3^; 2 rats/cage × 4, n = 8) or in EEE cages ((a), right panel and (b–d); EEE cage: 30″ × 20″ × 18″ = 10,800 in^3^; 4 rats/cage × 2, n = 8). The EEE housing further stimulated physical activities of rats by providing water (red arrowhead in (d) and food (yellow arrowhead in (d)) at separate ends and on different floors, in addition to the tunnels and stairs in a markedly enlarged space to augment walking, exploring, and playing (b–d). The EEE cage also increased opportunities for rats to socialize due to a bigger habitat group (e.g., 4–6 rats/per EEE cage versus 2 rats/regular cage). Quantified data (collected at 11AM, 4PM, and 7PM) showed that the EEE housing doubled or tripled the ambulation distance of the rats compared to the conventional caging ((e); blue: traditional group; purple: EEE group).
