# Supplementary figures and images for: T12-L3 Nerve Transfer-Induced Locomotor Recovery in Rats with Thoracolumbar Contusion: Essential Roles of Sensory Input Rerouting and Central Neuroplasticity
Source: Cells. 2023 Dec 8;12(24):2804. doi: 10.3390/cells12242804 (PMC10741684; doi:10.3390/cells12242804)

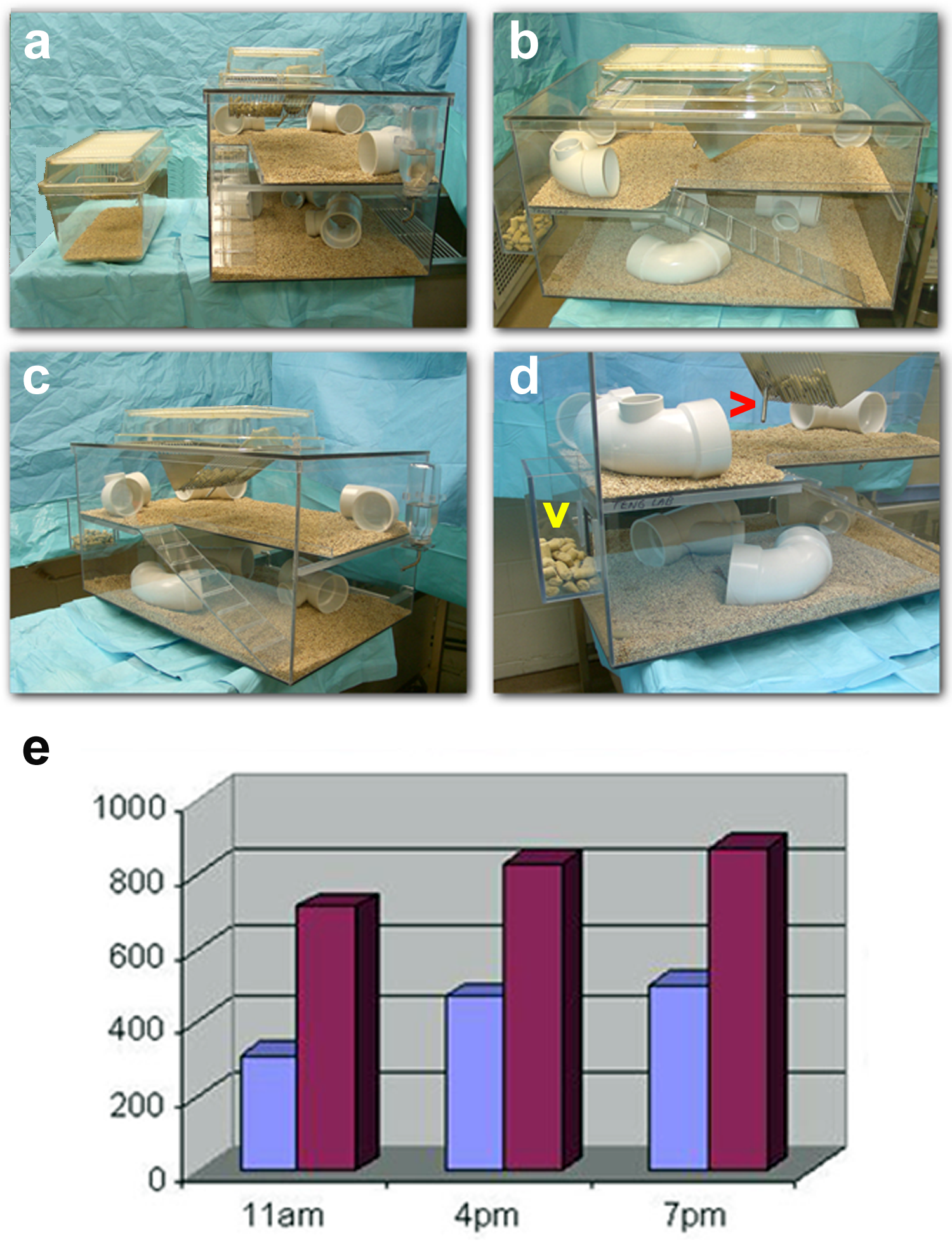

Supplement: Supplementary file 1 [file cells-12-02804-s001.zip › cells-2679560-supplementary.tif]
